# Water and sectoral policies in agriculture–forest frontiers: An expanded interdisciplinary research approach

**DOI:** 10.1007/s13280-021-01555-5

**Published:** 2021-04-21

**Authors:** Chris Seijger, Daniela Kleinschmit, Dietrich Schmidt-Vogt, Muhammad Mehmood-Ul-Hassan, Christopher Martius

**Affiliations:** 1 Wageningen University, PO Box 414, 6700 AK Wageningen, The Netherlands; 2 University of Freiburg, Tennenbacherstr. 4, 79106 Freiburg in Breisgau, Germany; 3 Faculty of Environment and Natural Resources, Albert-Ludwigs-Universität Freiburg, Tennenbacherstr. 4, 79106 Freiburg, Germany; 4 World Agroforestry (ICRAF), PO Box 30677, Nairobi, 00100 Kenya; 5 Center for International Forestry Research (CIFOR) Germany gGmbH, Charles-de-Gaulle-Strasse 5, 53113 Bonn, Germany

**Keywords:** Afforestation, Forest, Policy coherence, Pendulum swing, Regime shift, Water resources

## Abstract

Major land use changes such as deforestation and restoration influence water resources in agriculture–forest landscapes. Changes are observed in water flows, groundwater infiltration, water quality and rainfall. Interdisciplinary water–forest research has unravelled biophysical parts of the interplay that influences forest and water resources. In this Perspective paper, we propose an expanded interdisciplinary research approach to study water and policies in agriculture–forest frontiers. The approach differs in four important aspects from previous ones: (i) a conceptual ‘frontier’ understanding; an analytical focus on (ii) agriculture and (iii) policy–water linkages; (iv) empirical attention to northern and southern countries. The approach is put into practice with the “Pendulum” framework, with interventions and the agriculture–forest frontier oscillating over time between exploitation and restoration. Through the approach, a better understanding will be provided on the dynamic interplay of water and policies in oscillating agriculture–forest frontiers, with changing outcomes for people and environment.

## Water and forests, a dynamic and longstanding research field

Alexander von Humboldt was among the first to write about linkages between water, forests and agriculture when he visited the Aragua Valley in Venezuela 1799–1800. “When forests are destroyed, as they are everywhere in America by the European planters, the springs are entirely dried up, or become less abundant. (..) the waters falling in rain are no longer impeded (…) during heavy showers they bear down the loosened soil, and form those sudden inundations, that devastate the country” (in Wulf 2015, p. 57). In the US, Marsh (1907) also extensively covered water–forest relations under deforestation. Policy responses to problems related to water–forest linkages followed in the nineteenth century. In Switzerland, France and Germany, forest laws were enacted involving reforestation and restoration of alpine areas (Mather and Fairbairn 2000). The examples show that early water–forest research was undertaken in response to water problems that arose from land use changes in agriculture–forest landscapes.

The sweeping observations on water–forest linkages from the nineteenth century were increasingly tested in the twentieth century. An interdisciplinary research field emerged where foresters and hydrologists empirically investigated water–forest linkages in a complex pattern of influencing factors (climate, geology, soil depth, slope, forest cover percentage, tree species and age). The US Forest Service was in 1910 the first to conduct a pairwise comparison of watersheds where the effects of an intervention (e.g. clear felling) on water components (stream flow and sediments) were examined in one watershed in comparison with a reference watershed (Amatya et al. 2016). The paired comparison became an important technique, applied in hundreds of studies worldwide. In addition, forest hydrology research sites were developed in Europe and China (Mai 1975; Zhang et al. 2004; Birkinshaw et al. 2014). Insights of water–forest studies were synthesised at the start of the twenty-first century. Bonell and Bruijnzeel (2005) concluded that in the tropics, forest clearing increases stream flows during the dry season, affects small–medium floods locally, and does not lead to significant reductions in rainfall. Van Dijk and Keenan (2007) concluded that afforestation on agricultural land is likely to reduce average streamflow, groundwater recharge, low flows (except in degraded environments), shallow landslides and local flash floods. Amatya et al. (2016) summarised the current state of knowledge of hydrological processes in forests across the world. A rather new topic is the dynamic role of forest lands in recycling rainfall (van Noordwijk and Ellison 2019).

With all the knowledge gained, urgent calls remained for enhanced understandings of water–forest linkages and embedment in policies, as deforestation continues at a global scale, in parallel with further degradation of surface and groundwater bodies (Crowther et al. 2015; de Graaf et al. 2019). The water–forest theme has been picked up by global commitments and reforestation NGOs.^1^ Water has become one of the central arguments to conserve and restore forest ecosystems. In spite of this, and while good case practices have been reported (e.g. UNECE/FAO 2018), several studies conclude that linkages between water and forest remain underrepresented in decision-making (Ellison et al. 2017; Creed and van Noordwijk 2018).

The brief overview shows that interdisciplinary water–forest research has been undertaken mostly to understand linkages between forest and water systems under changing forest cover and land use (e.g. van Dijk and Keenan 2007; Amatya et al. 2016; Jones et al. 2017). We conclude from the above overview that water–forest research has been developed along interdisciplinary lines (forest hydrology, geography, ecohydrology, see references in this section) largely within the confines of a biophysical perspective of water resources and forests. One of the main purposes of this paper is to widen the perspective by sketching the contours of an interdisciplinary research field that goes well beyond water and forestry by including an agriculture and policy perspective.

Specifically, this paper intends to broaden the water–forest research field by proposing an expanded, interdisciplinary research approach that combines conceptual, analytical and empirical aspects that up until now have remained largely unaddressed in water–forest studies. *Conceptually*, the approach adopts a ‘frontier’ perspective to comprehend the exploration, exploitation and contestations over forest and water resources in agriculture–forest landscapes (Thurner 1893; Brando et al. 2013), going beyond the focus of biophysical studies on land use change or pairwise comparisons (e.g. Amatya et al. 2016). *Analytically,* the approach includes both agriculture as the single biggest driver of deforestation (Maertens et al. 2006; Tanentzap et al. 2015) and the linkage of sectoral policies with water resources, in contrast to biophysical studies which tend to treat agriculture and policies as boundary phenomena for forest–water systems (e.g. van Dijk and Keenan 2007). *Empirically*, the approach focuses on de- and reforestation hotspots in the Global North (Garcia-Chevesich et al. 2017) and in the Global South, unlike most other water–forest studies that focus only on deforestation in the Global South (e.g. Bonell and Bruijnzeel 2005; Brando et al. 2013).

These four aspects (‘frontiers’, agriculture, linkage to policies, Global North and South) are further explained in Section “Conceptual, analytical and empirical aspects of an expanded, interdisciplinary approach for water–forest research”. The expanded approach is interdisciplinary with linkages between social and environmental sciences. These linkages can be made in different ways. Section “Putting the expanded interdisciplinary research approach into practice” therefore explores how the approach can be put into research practice.

## Conceptual, analytical and empirical aspects of an expanded, interdisciplinary approach for water–forest research

### Conceptual: Water in the agriculture–forest frontier

For social scientists, conceptual framings (or analytical lenses) are important while the lenses clarify the perspectives and assumptions to study humans, their interactions and actions in society and the wider natural environment. One lens that has become popular in relation to water is ‘nexus’, be it in the water–forest nexus (Springgay et al. 2019), the water–energy nexus, or the water–energy–food nexus (Terrapon-Pfaff et al. 2018). A nexus lens emphasises interlinkages between water and other domains, with choices and actions in one domain affecting the others. The nexus lens is frequently used in international policy networks to link the Sustainable Development Goals, and in modelling approaches to explore future developments and trends of nexus domains in an integrated way (Bleischwitz et al. 2018).

Whereas the nexus lens is relatively abstract or remote from day-to-day impacts of a nexus on people and environment (e.g. connections between nexus domains, future modelling of water–energy–food trends), a frontier^2^ perspective embeds human-driven changes, interests, politics and conflicts around forests and water resources (e.g. deforestation, water pollution, dropping water tables) in the specific geographical context of an agriculture–forest frontier. An agriculture–forest frontier is defined as a dynamic physical and non-physical border area between agricultural and forest lands, which changes over time due to human interventions and biophysical processes. The frontier notion goes beyond changes in land use or water resources. It draws attention to exploration and exploitation of agriculture–forest landscapes and the conflicts that emerge over who obtains the benefits of natural resources (Brando et al. 2013; Coy et al. 2016). Water resources are not lost (yet may change location or shift between users) but remain in the hydrological cycle. It is thus a matter of who obtains the water benefits, and who loses or gains when water resources shift from one user to another. The frontier framing further recognises that agriculture–forest landscapes are contested areas with conflicting policies, values and ideas on how to manage or exploit water and land resources. In addition to the above-mentioned social implications of the frontier notion, there are geographical and biophysical implications. Though the term “frontier” is commonly associated with a sharp border (the notion of a landscape bisected by a neatly drawn dividing line), a frontier is a transition space, diffuse and dynamic both in time and extent, wherein human pressures and various land use forms (agriculture, forestry, protected forests, agroforestry) affect water resources (Bryant et al. 1997; Agrawal et al. 2014; Coy et al. 2016; van Noordwijk 2019).

In sum, a frontier conceptualisation is more adequate in fleshing out the allocations of and conflicts over land and water resources, whereas a nexus lens emphasises connections between water and related domains with limited attention for conflict (e.g. Bleischwitz et al. 2018; Springgay et al. 2019). Combinations between the two are possible, and may bring together the best of both perspectives. Nexus relations like agroforestry (van Noordwijk 2019) or the positive effects of reforestation on groundwater recharge or enhanced rainfall (Ellison et al. 2019) could be studied in frontier landscapes with linkages to prevailing policies. As an example, agroforestry is often regarded as a win–win solution for agriculture and forests. Yet in agroforestry areas in the North China plain, groundwater levels are lowered by irrigation of crops, resulting in an accelerated decline of agroforestry (Liu et al. 2020).

### Analytical: Inclusion of agriculture in water–forest relationships

Agriculture is an important analytical topic because expanding agriculture is the single biggest driver of land use change (IPBES 2019), affecting forests and related water systems. Since the beginning of permanent agriculture, 31% of the total forestland disappeared (Crowther et al. 2015), and agriculture accounts for 70–95% of this loss (Tanentzap et al. 2015). Logging, rural and urban expansion are other factors that alter forest land use. Agricultural expansion which starts in the forest margins and then moves deeper into forests is a characteristic feature of agriculture–forest frontiers. Whether or not trees alter water flows (Reynolds and Thompson 1988; van Dijk and Keenan 2007), a consensus has emerged that forests in various degrees of degradation and fragmentation have a central role in water cycling and protecting water quality (Bonell and Bruijnzeel 2005; Ellison et al. 2017). In addition to the effect of forests on run-off (river flow) and infiltration, there is increased recognition that evapotranspiration of forests represents an important water flux over long distances in the higher atmospheric strata, e.g. Amazon deforestation is related to reduced rainfall in the Amazon basin (Spracklen and Garcia-Carreras 2015). A change in land use from forests to agricultural land thus comes with changes in water quantity (e.g. evapotranspiration, infiltration, rainfall, water availability) and water quality (e.g. inflow of pesticides, fertilisers), in surface- and groundwater (Bruijnzeel et al. 2005; Maertens et al. 2006; Brando et al. 2013; Spera et al. 2016; Abbott et al. 2019).

Yet to date, agriculture has received limited attention in water–forest studies. Agricultural scientists mostly study water resources on agricultural fields (e.g. irrigation and drainage), whereas forest scientists do the same in forests (forest hydrology). Interdisciplinary studies that do integrate agriculture, forests and water resources cover agroforestry (van Noordwijk 2019; Liu et al. 2020) and human modifications in the global water cycle (Ellison et al. 2017; Abbott et al. 2019). Despite the obvious influence of agriculture on forests and related water resources, and the accelerated agricultural expansion in forest areas in South America, Africa and Asia (Ordway et al. 2017), research gaps about the interactions remain. How do water–forest relationships change under agricultural expansion and intensification, for instance for soybean and rainfall recycling in the Amazon (Salati et al. 1979)? What is the degree and speed of recovery in water resources (surface water, groundwater) under reforestation on former agricultural fields?

### Analytical: Linking water resources with sectoral policies

The impact of policies on water resources is relevant to understand water–forest systems. Streams were drying up when the royal forests were cut down after the French Revolution (Ford 2016)—and rising groundwater levels, stream formation and erosion are a result of contemporary deforestation for soy in central Argentina (Contreras et al. 2012). It is undisputed that formal and informal policies for land use (e.g. conservation, deforestation, afforestation) have an influence on forest cover, agricultural land use and hence on water resources. Studies furthermore show that agricultural and forestry policies are often incoherent and conflicting (Huttunen 2015; Tanentzap et al. 2015).

Policy influence is manifested in a process, stretching from policy formulation and approval, towards instruments to implement policies (e.g. concessions, licences, altering tenure rights) with effects on land use change (including conservation) and water resources. The pathway that links policies with water resources is not always direct or clearly visible (informal policies serving hidden agendas, inappropriate policy instruments, incoherent or conflicting policies for forest, agriculture and water, changed rainfall patterns, time lag, upstream interventions, or other human interference that influence water resources). Looking at how policies for forests, agriculture and water are implemented or not, or to a limited extent, can offer important insights into why water resources are changing under agricultural expansion, deforestation and reforestation. Recently, scholars have advocated for more integrated and coherent policies to sustain forests and water resources (Calder et al. 2007; Ellison et al. 2017; Creed and van Noordwijk 2018; Baulenas 2021).

Impacts of policies on water resources are mostly studied under the headers of water policy, water governance or integrated water resources management (Pahl-Wostl 2015; Kochskämper et al. 2016; Zwarteveen et al. 2017). Yet, interdisciplinary studies on the connection between people’s interventions (e.g. policies) and a changing state of water resources in forested landscapes remain a largely unexplored field of research with notable exceptions (e.g. Bonel and Bruijnzeel 2005; Brando et al. 2013; Hasselquist et al. 2020). Research can address the ways and modes of governance through which policies are formulated, and the subsequent linkages between sectoral policies (e.g. for agriculture, forests, water supply) and policy impacts on water resources under conservation, deforestation, and reforestation regimes. Reforestation is becoming particularly relevant in a changing global policy context (see global commitments in footnote 1), where reforestation efforts are undertaken to restore forests and water systems. Assessing the impact of reforestation policies and projects on water resources is challenging. There is not one generic relationship of the kind of ‘more trees are always better for water’; scientists disagree whether reforestation generally reduces stream flows (van Dijk and Keenan 2007) or whether stream flow reductions cannot be linked to reforestation as water-intensive trees (e.g. Eucalyptus and Pinus) and short-term monitoring produce distorted results (Cunningham et al. 2015; Filoso et al. 2017).

### Empirical: Frontiers in the Global North and Global South

A focus on de- and reforestation in the Global North and Global South is the fourth and last element in the expanded interdisciplinary approach to research on water in agriculture–forest frontiers. The agriculture–forest frontier concept has been mostly applied to the exploration and exploitation of the last remaining primary forests (Bryant et al. 1997) and secondary forests (van Vliet et al. 2012). Frontier case studies tend to focus on the “Global South”^3^ as major deforestation hotspots lie in the Amazon, Congo and Indonesia (Rösler 2001; Maertens et al. 2006; Brando et al. 2013; Coy et al. 2016). Yet the frontier notion also pertains to the Global North. Recent deforestation in Spain and Romania led to water quantity and water quality problems (Garcia-Chevesich et al. 2017), while in California and Australia conflicts about water–deforestation–agriculture remain unresolved (Charbonneau and Kondolf 1993; Creed and van Noordwijk 2018). There is thus a research gap how water–forest relations change across northern and southern countries under the influence of agriculture and sectoral policies.

What is more, acknowledging the context wherein reforestation initiatives operate, the direction of a frontier may revert when priority is given to restoring forests and water resources over agricultural land use. These new frontier directions are diverse in their dynamics, locations and water issues. For instance, riparian forests of the river Rhine in Germany and France dried out due to straightening of the river. They were converted to agricultural land with less than 10% of the original riparian forest remaining (Deiller et al. 2001; Dister et al. 1990). Current efforts to restore the riparian forests along the Upper Rhine are constrained by the fact that large parts of the former forest areas have become hydrologically disconnected from the river–groundwater system. In Kenya, infrastructural developments and demands for fuelwood have desiccated the montane forests that are instrumental for water provisioning (they are also known as Kenyan water towers). In 2000–2010, deforestation of an estimated 50.000 hectares resulted in reduced river flows in the dry season, and increased wet season flows that eroded fertile soils. Efforts to restore forests and water resources are undermined by illegal deforestation activities. Moreover, the 10.000 hectares of restored forest are considerable smaller than the deforested area (UNEP 2012).

## Putting the expanded interdisciplinary research approach into practice

Having outlined the main aspects of the expanded interdisciplinary research approach, what sort of research could it generate? Ideally, the four components—frontiers, agriculture, policy–water linkages, Global North and South—are covered within one research project. However, studies may also address a single case. This section explores how the interdisciplinary research approach can be put into practice through a case study design, methods for hydrological and policy studies, options for interdisciplinary research, and working hypotheses.

### Case study design and disciplinary methods

The variety in agriculture–forest landscapes and the various phases of exploration, exploitation and restoration in frontiers illustrate the importance of studying very different cases to understand the changing state of water resources in agriculture–forest frontiers. A *most-different case research design*—in which very different agriculture–forest frontiers are studied and compared (after Steinberg and VanDeveer 2012)—enables a study into the phenomenon of interest—the complex interplay of social and environmental factors that affects water resources in dynamic agriculture–forest frontiers. Cases share this phenomenon and differ with respect to the social-economic and biophysical contexts in different regions. Table 1 summarises criteria for case selection that inform such a most-different case research design. A comparison of empirical studies in different frontiers can lead to a typology of archetypal water–forest–policy interplays in different frontier contexts, wherein each archetype clusters (different) influential factors that have significantly influenced the water–forest interplay. The typology of water–forest–policy interplays does not have to be created through one major most-different case study, but can be built up through a meta-study, where single case studies, and comparative studies within a northern or southern context, are aggregated (after: Stewart 2012).

**Table 1:** Criteria for selecting most different agriculture–forest frontiers

Different criteria	Similar dynamics
Climate zones (e.g. boreal, temperate, sub-tropical, tropical)	In the past decades, large land conversions took place from forest to agriculture
Landscape types where forests and agriculture occur (e.g. plains, valleys, low to medium mountains)	Hydrological situations have been altered by land cover changes at different points in history, resulting in water problems for different water users, including the environment
Socio-economics (e.g. countries in different continents)	Presence of policies for agriculture, forest, water
Political economy (e.g. free-market economies, command economies, mixed economies)	Efforts are, or have been, undertaken to regenerate forest and water resources

**Fig. 1:**
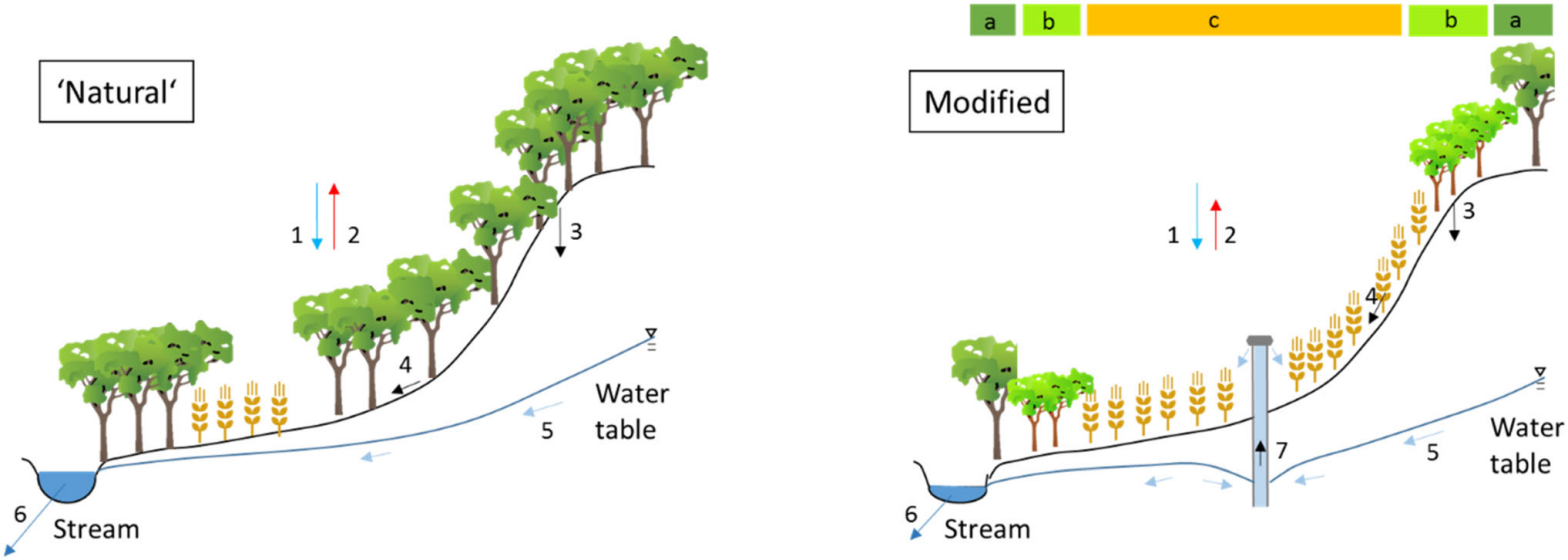
Conceptualisation of large-scale changes in hydrology and land use in an agriculture–forest frontier, shown are a more natural (left) and a modified situation (right). Water fluxes (1–7): 1. Precipitation, 2. Evapotranspiration, 3. Infiltration, 4. Overland flow, 5. Groundwater flow, 6. Stream flow, 7. Groundwater pumping. Right figure shows zones for representative research sites in remaining forest (**a**), newly planted forest (**b**), agricultural land (**c**)

For each case study, methods (highlighted in *italics*) must come from different scientific disciplines to unravel the complex interplay that influences changing water resources in agriculture–forest frontiers. Hydrological methods must quantify water flows, and determine how these are affected by land use changes in forest and agriculture. *Field data* on the different water fluxes must be obtained for different land covers (see Fig. 1 for different water fluxes and typical land use changes in an agriculture–forest frontier). Field data can be obtained through existing monitoring sites or (new) field campaigns. The obtained data could be fed into a *water accounting model* that indicates how for different representative research sites (a, b, c in Fig. 1) water flows (e.g. river flow, groundwater, infiltration, evapotranspiration, rainfall) are affected by sectoral withdrawals and land use change (Karimi et al. 2013). GIS data, satellite imagery and historical land use maps produce insights for trends in land use. The water accounting and land use data are input for a *spatially distributed hydrological model* that offers quantitative insights how water flows are affected by land use change in the agriculture–forest frontier.

Methods for policy studies have to focus on the conflicts over forest and water resources in an agriculture–forest frontier, the politics and political bargaining to formulate policies (Candel and Biesbroek 2016) and the (non-) implementation of sectoral policies (water, forest, agriculture) across multiple levels (international, national, sub-national, local) over time. A *synergy conflict analysis* entails a study of influential policy and planning documents. It reveals which policies and plans were influential in changing land use for the different land covers (a, b, c in Fig. 1), what were the major policy conflicts, and to what extent policies were coherent across sectors in achieving objectives of national governments. A p*olicy coherence* study could explore the consistency across policy objectives, instruments and implementation (Nilsson et al. 2012) for water, forest, and agricultural policies in the frontier under study.

### Two possibilities for interdisciplinary research

The proposed methods have at this point covered water resources and the politics and policies for water, forest and agriculture. The interdisciplinary linkages between water resources and sectoral policies must be made in shared case studies, and this can be done in different ways. Two options are explored in the remainder of this section.

The first option is to establish these linkages ex post. Although this may be the more common practice, it is relatively weak in interdisciplinary integration as it happens at the end of a research project. For instance, policy implementation could be studied for areas where hydrological data are available, water accounts can be created, and impacts of policy priorities on water resources can be assessed over time (Calder 2007; Hasselquist et al. 2020). A challenge for empirical analysis of hydrological change is monitoring, because the often too short monitoring period undermines the statistical proof of cause–effect relations in paired watershed studies. An alternative approach is to combine remote sensing with geographical information systems. Such interdisciplinary research generates insights into which policies have been influential in changing land and water resources over time in a particular agriculture–forest frontier. Or, when field data remain an issue, spatial–hydrological models could be used to run policy scenarios which explore implications of (proposed and alternative) policies for water and land use in an agriculture–forest frontier.

The second option is the use—ex ante—of an interdisciplinary analytical framework. Interdisciplinary integration is strong as it is built into a research project from the beginning. We propose such an interdisciplinary framework around the notion of a pendular move, a back-and-forward swing over time. The pendulum notion has been applied in hydrology—how a river basin moves between phases of agricultural expansion and ecosystem restoration (Kandasamy et al. 2014)—and in policy sciences—investigating movements between styles of policy making like neoliberalism and social-oriented capitalism (Wallace 2000). A pendular move describes the dynamic nature of forest–agriculture frontiers where changes in policies and land use swing back and forth spatially and temporally, among people and environment. It thus goes further than a forest transitions concept (Mather 1992; Meyfroidt and Lambin 2011) that mostly looks at increases in forest land, without distinguishing between natural forests and forest plantations, nor the underlying improved ecosystem services such as water (Zhai et al. 2017).

**Fig. 2:**
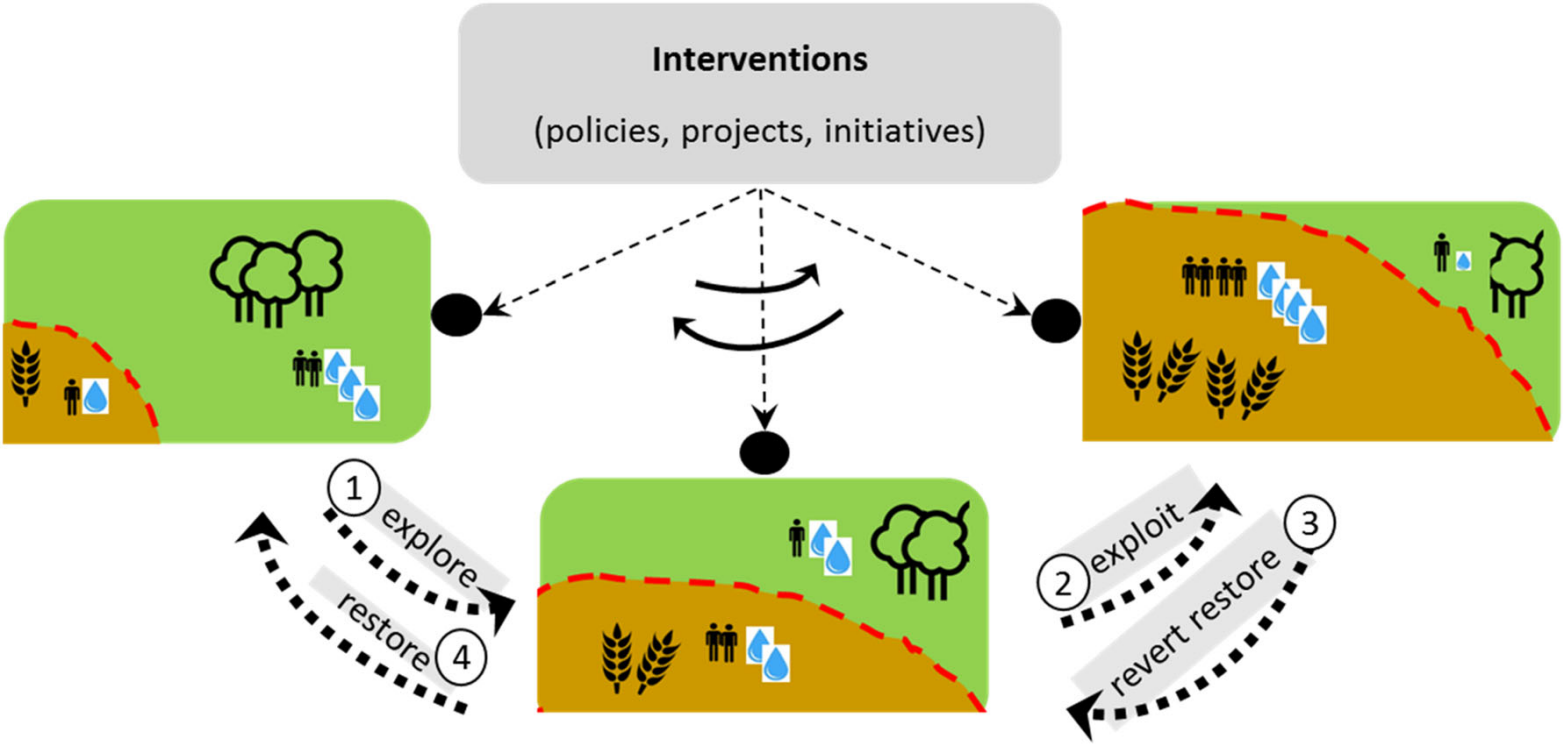
Pendulum framework to study pendular moves in actor’s interventions and land and water resources in the agriculture–forest frontier

The Pendulum framework is anchored in agriculture–forest frontier literature, specifically in studies that cover dynamic boundaries of agriculture–forest frontiers (Bryant et al. 1997; Agrawal et al. 2014; Coy et al. 2016); interventions of people to change and revert land use (Rudel et al. 2005; Torres-Salinas et al. 2016); linkages between water resources and land use (Bruijnzeel et al. 2005; van Dijk and Keenan 2007; Spera et al. 2016; Ellison et al. 2017); and changes in frontier water and land use in relation to inequality and biodiversity (Bryant et al. 1997; Rösler 2001; Delang 2002; Andersson et al. 2013; Brando et al. 2013). The Pendulum framework (Fig. 2) enables an interdisciplinary analysis into actors’ interventions and changing water resources in agriculture–forest frontiers, with interventions (e.g. policies, projects to implement policies) and the frontier moving over time like a pendulum with different outcomes for people and environment.

The Pendulum framework needs to be applied in case studies, to gain a better understanding of the dynamic and complex interplay of actor’s interventions and land and water resources. Pendulum analyses could cover a back-and-forth movement between different phases of exploration–exploitation–restoration (1–4 in Fig. 2). The analysis links natural resources with policies as quantitative hydrological analyses (how land/water use changed) are combined with qualitative narratives that describe driving factors (e.g. policies, politics and social struggles), their interplay and outcomes. It typically may take decades before a pattern of land use change over time can be discerned. Studies should therefore cover past developments, include long time series of data (water resources, land use, policies), and should have (access to) a long-lasting and continuous monitoring component (preferably 5–15 years, if not longer).

In sum, Pendulum analyses discuss *linkages* between sectors (e.g. water, forest, agriculture) and across scales that find expression in conflicts over policy formulation and implementation; the policy influence on *interventions* to restore land and water resources; and the *extent* to which actor interventions and their implementation across levels are observable in water resources and land use. While interventions in agricultural expansion and reforestation will be key factors determining how the pendulum swing steers changes in land use and different types of water resources (ranging from surface water to groundwater and precipitation recycling), other driving factors like rural–urban expansion, mining and logging can also play important roles. Although linkages between policies, implementation, land use change and water resources are difficult to discern, empirical policy studies for the Brazilian Soy Moratorium (Nepstad et al. 2019) and European Water Framework Directive (Giakoumis and Voulvoulis 2018; Hasselquist et al. 2020) show that this can be done.

### Working hypotheses

The expanded interdisciplinary research approach broadens the water–forest research field as it supports research in an agriculture–forest frontier conceptualisation that links sectoral policies to water resources in a spatial–temporal context. The approach can be used to test working hypothesis that are relevant in different academic and policy debates (shown in Table 2).

**Table 2:** Working hypotheses fitting the expanded interdisciplinary research approach, to be tested in empirical studies

No	Working hypothesis	Science and policy debate
1	Reversing land cover changes along the frontier is severely constrained due to substantial changes in hydrology	Hydrological regime shifts in the Anthropocene and the contribution of human alterations (e.g. Gordon et al. 2008; Rockström et al. 2014)
2	In the agriculture–forest frontier, there is little policy coherence across local–national–international levels for the policy sectors of water, forest and agriculture	Calls for policy coherence and empirical analyses of it in northern and southern contexts (e.g. Hogl et al. 2016; Ellison et al. 2017)
3	When studying agriculture–forest frontiers over time, a back-and-forth move can be observed in water resources, land use and policies	Integrated analyses how water resources are affected in frontiers (e.g. Brando et al. 2013) Pendular frameworks across hydrological and policy sciences (Wallace 2000; Kandasamy et al. 2014)

## Concluding thoughts

Water–forest linkages represent a longstanding research field that is receiving renewed attention in academia and policy (e.g. Ellison et al. 2017; Creed and van Noordwijk 2018; UNECE/FAO 2018; Springgay et al. 2019; Baulenas 2021). Whereas the interdisciplinary research field has made tremendous advancements on how water resources are biophysically linked to forests (e.g. Bonell and Bruijnzeel 2005; van Dijk and Keenan 2007; Amatya et al. 2016; van Noordwijk and Ellison 2019), major interdisciplinary research gaps remain regarding the complex interplay of factors that lie beyond the boundaries of biophysical inquiries, and which influence forests and water resources dynamically over time. This paper has laid the foundations for an expanded interdisciplinary research approach where agriculture and forest influences on water resources are jointly studied, with research that adopts a frontier perspective (conceptual), includes agriculture (analytical) and links water resources with sectoral policies (analytical), across agriculture–forest frontiers in northern and southern countries (empirical). Furthermore, the type of research to be conducted under this novel approach has been unveiled with a most-different case study research design, possibilities for single case studies and interdisciplinary research, the Pendulum analytical framework, and relevant working hypotheses.

But has this sort of interdisciplinary research not been adopted already? Unfortunately, not on a large scale. Despite increased attention for forest restoration potential at a global scale (e.g. Bastin et al. 2019), or hydrological processes in dynamic forest–agricultural landscapes in South America (e.g. Jones et al. 2017), the impact of policies on land use and water resources have been hardly covered. An expanded research approach is thus relevant and timely as it sketches the contours of an interdisciplinary water–forest research field that produces insights into the driving forces, human interventions, and outcomes for water resources and people in agriculture–forest frontiers. Such enhanced empirical insights have immediate practical use in policy and planning discussions as they indicate how water concerns can be integrated in cross-sectoral policies, policy instruments and land use strategies in dynamic, contested agriculture–forest frontiers around the world.

## Footnotes

1 Global commitments: Bonn Challenge 2011, FAO Forest and Water Action Plan 2015, AFR 100 African Forest Landscape Restoration Initiative, Trillion Tree Partnerships 2018. NGOs: WeForest, Ecosia, TREE AID.

2 The frontier concept was introduced by Thurner (1893) to describe the diverse frontiers through which colonisation of the “Great West” landscapes and Indian cultures occurred.

3 We acknowledge that the North–South distinction is overly simplistic (Therien 2010). We nonetheless use it to make the larger point that a study on changing water resources in dynamic agriculture–forest landscapes is not only relevant for specific regions in the world.
